# Is there any link between vitamin D deficiency and vasovagal syncope?

**DOI:** 10.1002/joa3.12309

**Published:** 2020-02-10

**Authors:** Songül Usalp, Hatice Kemal, Ümit Yüksek, Belma Yaman, Aziz Günsel, Oğuzhan Edebal, Onur Akpınar, Levent Cerit, Hamza Duygu

**Affiliations:** ^1^ Department of Cardiology Near East University Faculty of Medicine Nicosia Cyprus; ^2^ Department of Clinical Biochemistry Near East University Faculty of Medicine Nicosia Cyprus

**Keywords:** head‐up tilt table test, syncope, vitamin D deficiency

## Abstract

**Background:**

This study aimed to investigate serum 25[OH]D levels between patients with vasovagal syncope (VVS) diagnosed with head‐up tilt table test (HUTT) and age‐matched healthy people.

**Methods:**

The study included 75 consecutive patients (32.3 ± 10.7 years), who presented with syncope and underwent HUTT and 52 healthy controls (32.9 ± 14.1 years). HUTT patients were divided into two groups according to whether there was syncope response to the test. Patients underwent cardiac, psychiatric, and neurological investigation. Serum 25[OH]D levels were measured by chemiluminescent microparticle immunoassay method.

**Results:**

There was no difference between the two groups in terms of age, gender, body mass index (BMI), echocardiographic findings (*P* > .05). Mean serum 25[OH]D (24.5 ± 6.3 vs 20.1 ± 8.8 ng/mL, *P* = .003) and vitamin B12 levels (436.4 ± 199.2 vs 363.1 ± 107.6 pg/mL, *P* = .009) was lower in syncope patients when compared to the control group. In correlation analyses, syncope was shown as correlated with the vitamin D (*r* = −264, *P* = .003) and vitamin B12 levels (*r* = −233, *P* = .009). But, multivariate regression analyses showed that only vitamin D increased risk of syncope [OR: 0.946, 95% CI (0.901‐0.994)]. There was no difference in terms of age, gender, BMI, echocardiographic findings between the in HUTT positive (n = 45) and negative groups (n = 29). Only vitamin D level was significantly lower in HUTT positive group (17.5 ± 7.7 vs 24.4 ± 9.1 ng/mL, *P* = .002). There was no difference among in the vasovagal subgroups in terms of vitamin D level and other features.

**Conclusion:**

Vitamin D and B12 levels were reasonably low in syncope patients, but especially low Vitamin D levels were associated with VVS diagnosed in HUTT.

## Graphical abstract

Various diseases play a role in the etiology of syncope. In our study, we investigated whether there is a relationship between vitamin D and syncope. We found that the frequency of vasovagal syncope was increased especially in female gender and vitamin D levels below 20 mg/dL.

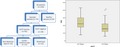

## 1 INTRODUCTION

Syncope is a transient loss of consciousness because of decreased blood flow to the brain and usually improves spontaneously. However, the prevalence and incidence of syncope vary according to population, vasovagal syncope (VVS) is one of the most common causes, it constitutes approximately half of the patients who admit with loss of consciousness.^1^, ^2^

VVS is often associated with emotional stress, pain, fear, prolonged standing, and crowded environments.^2^ The etiology of VVS syncope is because of interaction involving in the autonomic nervous system, which causes the predominance of the parasympathetic system and consequently development of bradycardia and hypotension.^3^

The initial assessment of VVS begins with history, physical examination and electrocardiography (ECG), followed by risk stratification and additional diagnostic tests, that is, blood test, cardiac imaging (transthoracic echocardiography), stress test, cardiac monitoring (in‐hospital telemetry, trans telephonic monitor, external loop recorder, patch recorder, mobile heart polyclinic telemetry), electrophysiological study (for the evaluation of selected patients with suspicious arrhythmic syncope), head‐up tilt table test (HUTT), carotid sinus massage, neurological test, and psychiatric evaluation.^1^

Although the prognosis of VVS is generally good, mortality is two‐fold increased in cardiac syncope (arrhythmias, structural heart disease).^4^ Treatment of syncope varies according to the underlying disease and aims to reduce symptoms by training and tune.^5^

The deficiency of serum 25‐hydroxyvitamin D (25[OH]D) is one of the underlying causes of a variety of disorders, including coronary artery disease, arrhythmias, neurological diseases, and cancers.^6^, ^7^, ^8^, ^9^ So far, there have been a large number of studies in the literature analysing the association of serum 25[OH]D and cardiovascular autonomic dysfunction, but studies involving the evaluation of the syncope with HUTT are very limited.^10^, ^11^, ^12^

In our study, we aimed to compare serum 25[OH]D levels between patients with VVS diagnosed with HUTT and age‐matched healthy people. In the second step of the study, the patients who developed syncope during the HUTT test and who did not develop syncope were compared.

## 2 METHODS

**Figure 1:**
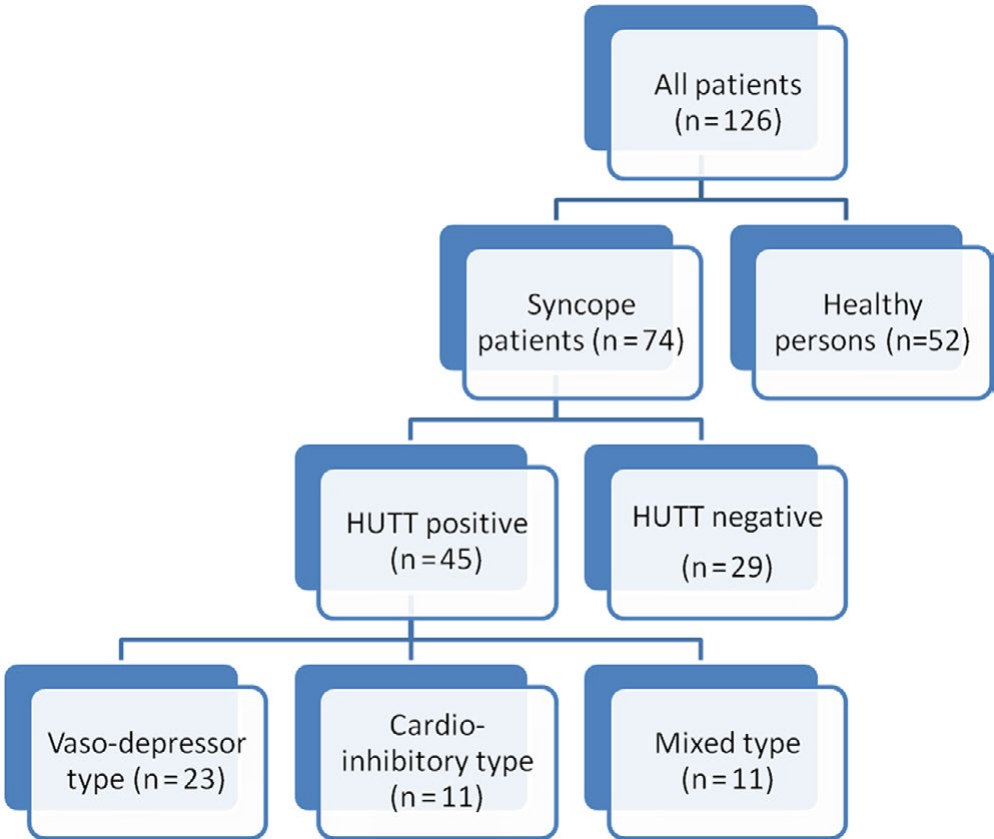
Flowchart of the number of patients included in the study

The study was a single‐center, retrospective study and data were obtained from patients who were admitted with syncope and had HUTT performed between 2016 and 2019. Fifty‐two consecutive healthy persons and 75 syncope patients were included in the study, and syncope patients were categorised based on their HUTT results (positive, n = 45 vs negative, n = 29) (Figure 1).

Patients with vagal syncope were divided into two groups as those with vitamin D levels above and below 20 ng/mL for determining factors underlying low vitamin D levels. And the subgroups of patients with VVS (vaso‐depressor, cardio‐inhibitor, and mixed type) were compared in terms of vitamin D levels and demographic characteristics, blood sample results, and echocardiographic findings.

Patients with parathyroid hormone disorders, chronic metabolic diseases, and any type of cancers, pregnancy, neurological and psychological diseases were excluded. We performed 12 lead ECG, conventional 2D echocardiography, extensive blood tests, carotid ultrasonography, 24‐hour rhythm and blood pressure Holter recordings, neurological and psychiatric evaluations, and HUTT to all of the patients.

### 2.1 Head‐up tilt testing protocol

Since it was first described by Kenny et al the HUTT has been performed in various protocols.^13^ The patients were taken to the procedure room with 12 hours of fasting. To prevent the patient from falling during the procedure, the patient was wrapped with a support strap. Venous cannulation was placed in the appropriate position on the patient's left or right arm. Before the test, the patient was stabilized in a supine position for 5 minutes.

In our clinic, the Italian protocol is used for the tilt test.^14^ After 20 minutes of passive phase, the provocation phase was initiated in patients without syncope; 300‐400 µg sublingual nitroglycerine was given after 20 minutes of the test.^14^ Throughout the procedure, patients were continuously monitored with a 3‐lead electrode. Arterial blood pressure was measured every 3 minutes and also re‐evaluated when the patient had symptoms. In patients who had positive HUTT, the classification of VVS was done according to the ESC guidelines: Vaso‐depressor type, cardio‐inhibitor type and mixed type.^15^

### 2.2 Serum 25(OH)D measurement method

After initial evaluation for syncope, blood samples were taken for the assessment of serum 25[OH]D, complete blood count, renal functions, liver functions, vitamin B12 level, thyroid function tests, and serum electrolyte levels after at least 8 hours of fasting. Serum 25[OH]D levels were measured by chemiluminescent microparticle immunoassay method (Architect, Abbott Diagnostics). After each sample was taken the standard protocol was applied: initially centrifuged at 3500 G at +4°C, then kept frozen at −20°C until analysis.

### 2.3 Echocardiography

In accordance with the recommendations of the American Society of Echocardiography (17), all patients underwent a transthoracic echocardiographic examination with a commercially available device (Vivid E9 system; General Electric Healthcare) using 4 MHz probe in the left lateral decubitus position. All conventional measurements (Left ventricular end‐diastolic and end‐systolic diameters, left atrium [LA] end‐systolic diameter, right atrium [RA] etc) were performed on the parasternal long‐axis and apical four‐chamber views. LV ejection fraction (EF) was calculated by Simpson's method.^16^

### 2.4 Statistical analysis

All statistical analyses were performed using SPSS version 20 (IBM Corporation). Quantitative variables reported as mean ± standard deviation (SD), and qualitative variables are expressed as a percentage (%). The baseline characteristics of the patients were compared using the Student's *t* test for continuous variables and the χ^2^ Pearson's test for categorical variables. In multivariate logistic regression, variables that were significantly associated with syncope were selected. Pearson's chi‐squared analysis was showed relationship for vitamin D deficiency in women gender and low BMI patients. One‐way ANOVA was used to assess the relationship between VVS subgroups and vitamin D level. A *P* value of less than .05 was considered statistically significant.

## 3 RESULTS

A total of 126 patients, 52 consecutive healthy persons (mean age of healthy group was 32.3 ± 10.7 years, 31 females [59.6%]), and 74 syncope patients (mean age of the patients was 32.9 ± 14.1 years, 55 females [73.3%]) were included in the study and syncope patients were categorized based on their HUTT results (positive, n = 45 vs negative, n = 30) (Table 1).

**Table 1:** Demographic characteristics of healthy people and syncope patients

Variables	Healthy persons (n = 52)	Syncope patient (n = 75)	*P* value
Age (years)	32.3 ± 10.7	32.9 ± 14.1	.821
Female gender, n (%)	31 (59.6)	55 (73.3)	.104
BMI (kg/m^2^)	24.1 ± 4.9	23.3 ± 2.8	.251
EF (%)	59.7 ± 1.4	59.2 ± 1.4	.520
LA (mm)	34 ± 0.4	33 ± 0.8	.850
RA (mm)	31 ± 0.3	32 ± 0.2	.122
Vitamin D (ng/mL)	24.5 ± 6.3	20.1 ± 8.8	**.003**
Vitamin B12 (pg/mL)	436.4 ± 199.2	363.1 ± 107.6	**.009**
TSH (mIU/L)	1.5 ± 0.7	1.8 ± 0.8	.128
Glucose (mg/dL)	90.6 ± 6.9	88.5 ± 8.1	.126
Creatinine (mg/dL)	0.7 ± 0.1	0.7 ± 0.1	.165
Sodium (mmol/L)	140.8 ± 3.4	139.7 ± 4.5	.250
Potassium (mmol/L)	4.0 ± 0.3	4.1 ± 0.3	.674
Calcium (mg/dL)	9.1 ± 0.4	9.0 ± 0.8	.476
Wbc (×10^3^/µL)	7.6 ± 1.7	7.2 ± 1.6	.147
Hb (g/dL)	13.5 ± 1.7	13.3 ± 1.4	.533
PLT (×10^3^/µL)	256.3 ± 51.4	257.8 ± 59.4	.979

Bold values indicates statistically significant results.

Abbreviations: BMI, body mass index; Hb, hemoglobin; HR, heart rate; kg/m^2^, kilogram/square meters; LA, left atrium; mg/mL, milligrams/milliliters; min, minute; mlU/L, milliunite/Liters; mm,millimeters; mmol/L,millimol/Liters; n, number of patients; ng/mL, nanogram/milliliters; pg/mL, picograms/milliliters; PLT, platelet; RA, right atrium; SBP, systolic blood pressure; TSH, tyhroid stimulant hormone; Wbc, white bloos cell.

Demographic, echocardiographic, and laboratory findings of healthy subjects and patients with syncope were compared (Table 1). There was no difference between the two groups when compared on BMI, gender, EF, RA, LA, thyroid‐stimulating hormone (TSH), glucose, creatinine, sodium, potassium, calcium, white blood cell, hemoglobin, and platelet values (Table 1).

However, vitamin D (20.1 ± 8.8 vs 24.5 ± 6.3 ng/mL *P* = .003) and vitamin B12 levels (363.1 ± 107.6 vs 436 ± 199.2 pg/mL, *P* = .09) were significantly lower in patients with syncope (Table 1).

Correlation analysis showed that syncope was associated with low vitamin D (*r* = −264, *P* = .003) and vitamin B12 levels (*r* = −233, *P* = .009) (Table 2). However, in multivariate regression analyses showed that only low vitamin D levels increased risk of syncope [OR: 0.946, 95% (0.901‐0.994)].There was no significant relationship between risk of syncope and vitamin B12 level [OR: 0.998, 95% (0.995‐1.000)] (Table 2).

**Table 2:** Correlation and regression analysis between syncope and age, gender, BMI, vitamin D, and vitamin B12 levels

Positive head‐up tilt testing	*r* value	*P* value	OR (95% CI of OR)	*P* value
Vitamin D (ng/mL)	−.264	**.003**	0.946 (0.901‐0.994)	**.028**
Vitamin B12 (pg/mL)	−.233	**.009**	0.998 (0.995‐1.000)	.069
Age (years)	.020	.821		
Female gender	.144	.106		
BMI (kg/m^2^)	.103	.251		

Bold values indicates statistically significant results.

Abbreviation: BMI: body mass index

**Figure 2:**
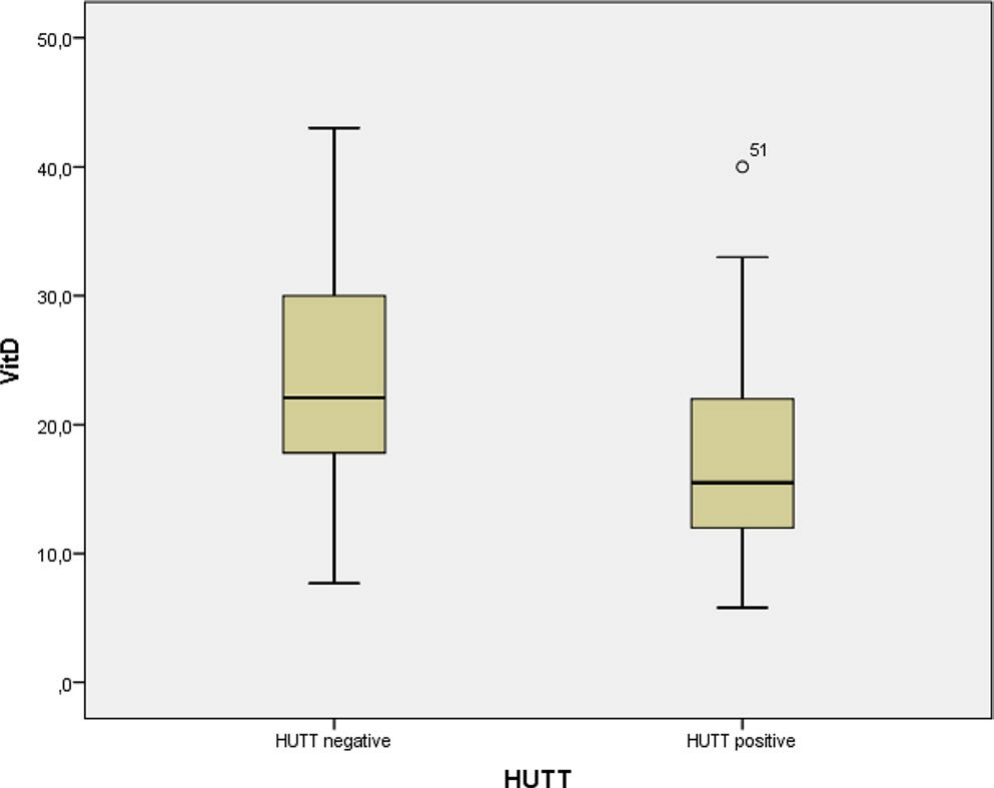
Vitamin D levels between head‐up tilt test (HUTT) positive and negative

Patients were divided into two groups as HUTT positive (n = 45) and negative (n = 29). There was no difference between the two groups regarding gender, age, BMI, blood values, TSH, vitamin B12 levels, and echocardiographic features (*P* > .05) (Table 3). Only, serum 25[OH]D levels were lower in the HUTT positive group than in the HUTT negative group (17.5 ± 7.7 ng/mL vs 24.4 ± 9.1 ng/mL, *P* = .002) (Table 3). The number of HUTT positive and vitamin D level <20 patients were 30 (75%), the number of HUTT negative and with vitamin D <20 ng/mL patients was 10 (25%) (*P* = .007).(Table 3, Figure 2).

**Table 3:** Patient characteristics of head‐up tilt testing positive and negative group

Head‐up tilt testing	Positive (n = 45)	Negative (n = 29)	*P* value
Age (years)	32.5 ± 13.7	33.5 ± 15.9	.973
Female gender, n (%)	35 (78.2)	18 (64.3)	.232
BMI (kg/m^2^)	23.4 ± 3.1	23.0 ± 2.6	.596
EF (%)	59.2 ± 1.6	59.1 ± 1.3	.357
LA (mm)	34 ± 2.4	33 ± 3.1	.715
RA (mm)	32 ± 2.5	31 ± 2.7	.141
Vitamin D (ng/mL)	17.5 ± 7.7	24.4 ± 9,1	**.002**
Vitamin D level <20 ng/mL, n (%)	30 (75)	10 (25)	**.007**
Vitamin B12 (pg/mL)	355.1 ± 124.6	378.5 ± 87.0	.367
TSH (mIU/L)	1.8 ± 0.8	1.7 ± 0.9	.648
Glucose (mg/dL)	87.0 ± 7.4	90.5 ± 8.1	.076
Creatinine (mg/dL)	0.6 ± 0.1	0.7 ± 0.1	.055
Sodium (mmol/L)	139.8 ± 2.0	139.7 ± 2.5	.931
Potassium (mmol/L)	4.1 ± 0.3	4.1 ± 0.2	.674
Calcium (mg/dL)	8.9 ± 0.9	8.9 ± 1.1	.476
Wbc (×10^3^/µL)	7.3 ± 1.6	6.9 ± 1.6	.339
Hb (g/dL)	13.3 ± 1.3	13.5 ± 1.8	.664
PLT (×10^3^/µL)	261.3 ± 55.8	249.9 ± 61.4	.132

Bold values indicates statistically significant results.

Abbreviations: BMI, body mass index; DBP, distolic blood pressure; Hb, hemoglobin; HR, heart rate; LA, left atrium; min, minute; PLT, platelet; RA, right atrium; SBP, systolic blood pressure; TSH, tyhroid stimulant hormone; Wbc, white blood cell.

VVS patients were categorized into three groups according to pathophysiology: Vaso‐depressor type (n = 23, 51.1%), cardio‐inhibitory type (n = 11, 24.4%), mixed type (n = 11, 24.4%). There was no significant difference in these subgroups concerning demographic characteristics, blood test results, serum 25[OH]D levels, and echocardiographic parameters.

## 4 DISCUSSION

In our study, we found that patients who were admitted to the hospital with syncope and underwent HUTT test had lower serum 25[OH]D and vitamin B12 levels than those who had healthy persons. However, only low levels of vitamin D were found to be significantly associated with syncope in advanced statistical analyses. Moreover, the patients who had syncope during HUTT test had lower vitamin D levels than those without syncope. Furthermore, no significant difference was observed among the VVS subgroups according to vitamin D level and other features.

Less exposure to sunlight, vegetarian individuals, pregnant or breastfeeding women, older adults, who have the gastrointestinal system and renal disease are under risk for vitamin D deficiency. In our study, syncope was found to be associated with low vitamin D levels regardless of gender and BMI values. In contrast to our findings, some studies investigating vitamin D levels in the general population found that vitamin D levels were lower in those with male gender and high BMI values.^17^ One of the important meta‐analysis results showed that low 25(OH)D levels (<20 ng/mL) were more prevalent in women, and vitamin D deficiency was more common in individuals with high BMI values.^18^

The role of serum 25[OH]D deficiency in VVS, can be summarized in several mechanisms: VVS is caused by an abnormal reaction of the autonomic system to various stimuli, such as a triggering event or upright position. In the event of sudden standing up, the inability to carry out the blood to the upper body causes stimulation on the aorta, carotid, and cardiopulmonary receptors.^19^

Decreased ventricular preload and severely volume‐consuming ventricles lead to elevated levels of catecholamines in patients with suspected VVS. It has been suggested that strong contractions of a volume‐emptied ventricle cause activation of cardiac C fibers (myelin‐free fibers in the atrium, ventricles, and pulmonary artery). Stimulation of these afferent C fibers leads to a “paradoxical’’ withdrawal of peripheral sympathetic tone and an increase in vagal tone, which in turn causes varying degrees of hypotension and bradycardia with syncope or presyncope.^3^

The active form of vitamin D is thought to be one of the main factors for the proliferation and development of vascular smooth muscle cells (VSMC), endothelial cells (EC), and immune system cells, which are the main cells in atherosclerosis and vascular elasticity.^20^ The Vitamin D receptor (VDR) present in these cells regulate VSMC relaxation and contraction by nitric oxide synthesis, and the calcium‐mediated pathways.^20^ Adverse effects of serum 25[D]D deficiency on VSMC and EC may contribute to syncope by causing deterioration of vascular function.

One of the reasons for syncope is impaired heart muscle functions. Mann et al showed a low vitamin D level (<20 ng/mL), causing cardiac autonomic dysfunction via repressing vagal balance.^12^ Thus, they have shown that this may increase the risk of cardiovascular disease. Also, Wang and Dobnig et al have shown that vitamin D deficiency increases the risk of cardiovascular disease and sudden cardiac death.^21^, ^22^ Studies also have shown that deficiency of vitamin D increases the tendency to heart failure.^21^, ^22^ As a result, vitamin D deficiency is an important independent risk factor for heart muscle dysfunction. In our study, the cardiac function measured via the echocardiography was normal in both syncope and healthy group.

Another factor that plays a role in the etiology of VVS is the disruption of neuronal conduction in the baroreflex mechanism. Vitamin D, which is also present in the central and peripheral nervous system, plays a role in maintaining the neurotrophic and neuroprotective effects of growth factors involved in the growth of nerve cells and neurotransmitter conduction.^23^ Because of the indirect effects of vitamin D on the central nervous system, smooth muscle cells and baroreceptor sensors, the risk of syncope may also increase in vitamin D deficiency.

When the syncope groups were divided into two groups according to the HUTT test and compared in terms of serum 25[OH]D level and other properties, no difference was found between each group. This finding reinforces our hypothesis that serum 25[OH]D deficiency may be one of the causes of syncope due to its effects on VSMC and EC, as well as on cardiac muscles and neurological transmission.^24^

The HUTT is an important non‐invasive, informative and simple tool that helps to understand the aetiology of syncope, in patients with suspected VVS and evaluation of autonomic dysfunction. In patients with syncope, the predictive value of the tilt table test is variable; in other words, tilt test was found to be positive in 51%‐56% of patients with atypical clinical features of reflex syncope, 30%‐36% of unexplained syncope, and 45%‐47% of patients with true cardiac arrhythmia.^2^ In our study, the percentage of patients diagnosed with vasovagal syncope by HUTT was slightly higher than in the literature.

The HUTT test was investigated in the management of syncope as well as in the diagnosis. But the HUTT has limited ability to assess the response of patients with syncope to treatment and prevent recurrent syncope.^5^

### 4.1 Study limitations

Limitations of our study were the small number of patients, retrospective characterization and being a single‐centre study. The same study was not performed before; for this reason, we could not compare the strength of the study. The importance of the study may be increased if we can determine that syncope numbers may decrease after vitamin D treatment in patients with lower vitamin D levels. Numerous studies are needed to understand the role of vitamin D in syncope.

## 5 CONCLUSION

To the best of our knowledge, our study is the first to investigate serum vitamin D levels in patients who had syncope with the HUTT and in patients who did not have syncope. We found that serum vitamin D levels were low in patients with syncope, especially in patients diagnosed with VVS by HUTT test. Therefore, measurement of serum vitamin D levels might be offered in VVS patients. In further studies, whether vitamin D supplementation will help improve VVS symptoms can be investigated. Larger randomised controlled trials in the future will shed light on association between vitamin D deficiency and syncope.

## CONFLICT OF INTEREST

Authors declare no conflict of interests for this article. Written informed consent was obtained from all patients before participating in the study

## AUTHOR CONTRIBUTION

All authors contributed toward the content, concept, writing, drafting and revisions of this manuscript and have approved the final version.
